# High Prevalence of Diabetes Among Indo-Guyanese Adults, Schenectady, New York

**DOI:** 10.5888/pcd10.120211

**Published:** 2013-03-28

**Authors:** Akiko S. Hosler, David S. Pratt, Kathryn A. Sen, Erin M. Buckenmeyer, Alexander Simao, Ephraim E. Back, Sanghamitra Savadatti, Jennifer L. Kahn, Glynnis S. Hunt

**Affiliations:** Author Affiliations: David S. Pratt, Kathryn A. Sen, Alexander Simao Jr, Glynnis S. Hunt, Schenectady County Public Health Services, Schenectady, New York; Erin M. Buckenmeyer, Sanghamitra Savadatti, University at Albany School of Public Health, Rensselaer, New York; Ephraim E. Back, Ellis Medicine, Schenectady, New York; Jennifer L. Kahn, Cornell Cooperative Extension of Schenectady County, Schenectady, New York.

## Abstract

**Introduction:**

The Indo-Guyanese population is the largest immigrant minority population in Schenectady, New York. A clinic-based study in Schenectady and surveillance reports from Guyana found high diabetes prevalence and mortality among Guyanese of Indian descent. No community-based study has focused on diabetes among Indo-Guyanese immigrants in the United States. We sought information on the prevalence of diabetes and its complications in Indo-Guyanese adults in Schenectady and compared it with the prevalence among non-Hispanic white adults in Schenectady.

**Methods:**

We administered a cross-sectional health survey at community venues in Schenectady in 2011. We identified diagnosed diabetes and its complications through self-reports by using a reliability-tested questionnaire. The final data set included 313 Indo-Guyanese and 327 non-Hispanic white adults aged 18 years or older. We compared the prevalence of diagnosed diabetes and diabetes complications between Indo-Guyanese and non-Hispanic whites.

**Results:**

Most Indo-Guyanese participants were born in Guyana, whereas most non-Hispanic whites were born in the United States. The crude prevalence of diagnosed diabetes among Indo-Guyanese participants and non-Hispanic whites was 30.3% and 16.1%, respectively. The age-standardized prevalence was 28.7% among Indo-Guyanese participants, significantly higher than that among non-Hispanic whites (14.5%, *P* < .001). Indo-Guyanese participants who had diabetes had a lower body mass index and were more likely to report poor or fair general health and eye or vision complications than non-Hispanic whites who had diabetes.

**Conclusion:**

Our study confirms the higher prevalence of diabetes in Indo-Guyanese adults in Schenectady. The higher prevalence of complications suggests poor control of diabetes. Excess burden of diabetes in this population calls for further research and public health action.

## Introduction

The Indo-Guyanese population is one of the fastest growing immigrant groups in North America (1) and the largest immigrant minority population in Schenectady, New York. The city is home to an estimated 8,000 Indo-Guyanese people (or 12% of the total population) and one of the most visible Indo-Guyanese communities in the United States. Most Indo-Guyanese trace their ancestry to the mid-1800s, when (East) Indian indentured servants were brought to Guyana, an English-speaking, continental West Indian country formerly known as British Guiana (1). Having very little intermarriage, this group is phenotypically and genotypically Indian and preserves its distinctive culture.

The Indo-Guyanese community is disproportionately affected by diabetes. A clinic-based study conducted in an academic hospital in Schenectady during 2004–2006 found an age-standardized prevalence of diabetes among Indo-Guyanese patients of 31.6%, twice that of whites and 65% higher than that of African Americans (2). Likewise, surveillance reports from Guyana indicated significantly higher diabetes prevalence and mortality among Guyanese of Indian descent (3–5), and New York City-based studies reported similar findings (6,7). The existing studies of Indo-Guyanese in the United States, however, are not population-based or are limited by secondary analyses of administrative data. No community-based study has focused on diabetes among Indo-Guyanese immigrants in the United States. The objective of this study was to ascertain the prevalence of diabetes, diabetes complications, and related health indicators in Indo-Guyanese adults in Schenectady and compare them with the same factors among non-Hispanic whites, the majority population in the community.

## Methods

We conducted a cross-sectional health survey from February to November 2011 in Schenectady, New York. Schenectady residents aged 18 years or older who understood and signed consent forms were eligible to participate in the survey. Power analysis showed that at least 280 Indo-Guyanese and 280 non–Indo-Guyanese respondents were needed to achieve a power of 90% for the expected prevalence difference. Because the US population census does not recognize Indo-Guyanese as a racial/ethnic category, and the Indo-Guyanese do not live in enclaves, we used convenience sampling for this study. We recruited respondents at 36 community locations and events: faith-based organizations (n = 8), community festivals (n = 6), worksites (n = 5), community meetings (n = 4), retailers (n = 4), schools (n = 3), health care facilities (n = 3), county government sites (n = 2), and a sporting event. Eleven of these locations and events catered primarily to the Indo-Guyanese community. A few Indo-Guyanese respondents were also recruited through referrals from community members and door-to-door visits. We monitored the age–sex distribution of Indo-Guyanese and non–Indo-Guyanese respondents and adjusted recruitment strategies to ensure that the sample had a similar age–sex distribution of the city’s adult population.

We developed a paper-and-pencil, self-administered, 42-item questionnaire in English. The questionnaire was structured at a 6th-grade reading level and used standardized questions from the Behavioral Risk Factor Surveillance System (8) and previously published studies (9,10). Test–retest reliability, assessed with a convenience sample of Indo-Guyanese and non–Indo-Guyanese (n = 12) found perfect agreement in 9 key variables, including age, sex, diagnosis of diabetes, and family history of diabetes, and excellent agreement (prevalence-adjusted bias-adjusted κ ≥80.0) in all other health-related variables. Trained research team members and trained members of Schenectady County’s Medical Reserve Corps and Women, Infants, and Children (WIC) staff provided assistance in completing the questionnaire, if requested by the respondent. The institutional review board of Ellis Hospital, Schenectady, reviewed and approved this study.

All information in this study was based on self-report. We identified respondents as Indo-Guyanese if they self-identified as Guyanese, indicated they were born in Guyana, or both, and they indicated their race as (East) Indian or Asian or had an Indian surname. Diagnosed diabetes was assessed by the question, “Has a health professional ever told you that you have diabetes?” We calculated body mass index (BMI) by using reported height without shoes (in inches) and weight (in pounds). Family history of diabetes was evaluated with the question, “Do/did you have a blood relative in your immediate family (mother, father, sisters or brothers) who has/had diabetes?” We assessed diabetes complications and comorbidities by lifetime diagnosis; diabetes-related hospitalization was assessed for the previous year.

Of 811 survey participants, 792 aged 18 or older remained in the data set after 12 duplicate responses and 7 ineligible respondents were removed. The 7 ineligible respondents had a Schenectady zip code but lived outside the city’s administrative boundaries. We found no significant differences in demographic characteristics between the removed and the remaining respondents. We computed sampling weights for the analysis. Indo-Guyanese and non–Indo-Guyanese samples were divided into 8 age–sex groups. We calculated an expected sample size for each cell by multiplying the total sample size by the fraction of the city’s age–sex distribution obtained from the 2010 census. The sampling weight was obtained by dividing the expected sample size by the actual sample size. We conducted univariate analysis, and data were weighted to reflect the population distributions in the study.

We computed prevalence of diagnosed diabetes, diabetes complications, and related health indicators for Indo-Guyanese (n = 313) and non-Hispanic whites (n = 327) with known diabetes status. Those in other racial/ethnic groups (n = 149) and whose diabetes status was unknown (n = 3) were not included. We used the *z* test for proportions to obtain the *P* values for differences in prevalence. The age-standardized prevalence of diabetes was computed to enable us to compare our results with the results of the earlier clinic-based study (2). We used the direct age-standardization method, which used the 2000 US population as a standard. We used SPSS-PC version 19.0 (IBM, Inc, Chicago, Illinois) and PEPI version 4.4 (11) for the analyses.

## Results

The mean age was 44.9 years for Indo-Guyanese and 47.4 years for non-Hispanic whites (Table 1). Most (95.9%) Indo-Guyanese were born in Guyana, whereas most (97.2%) non-Hispanic whites were born in the United States. Almost 60% of Indo-Guyanese had a high school diploma or the equivalent, and nearly half had an annual household income of less than $20,000. Approximately 25% of Indo-Guyanese were uninsured, compared with 6.4% of non-Hispanic whites. The mean BMI was similar in both groups.

**Table 1:** Weighted^a^ Characteristics of Indo-Guyanese and Non-Hispanic White Respondents, Schenectady, New York, 2011

Characteristic	Indo-Guyanese (n = 313)	Non-Hispanic White (n = 327)
**Age, y, mean (SD)**	44.9 (16.7)	47.4 (17.7)
**Female, %**	52.2	51.2
**Place of birth, %**		
United States	4.1	97.2
Guyana	95.9	0
Other	0	2.8
**Educational attainment, %**		
<High school graduate	31.3	2.6
High school graduate or equivalent	58.7	28.6
≥Some college	10.0	68.8
**Annual household income, %**		
<$20,000	49.1	12.9
$20,000-$49,999	40.1	22.9
≥$50,000	10.8	64.2
**BMI, mean (SD), kg/m^2^**	27.1 (4.8)	27.5 (5.8)
**Family history of diabetes, %**	57.1	43.4
**Health insurance coverage, %**		
Private insurance	38.4	68.5
Public insurance	35.5	25.1
Uninsured	26.1	6.4

Abbreviation: SD, standard deviation; BMI, body mass index.

a Data were weighted to reflect the population distributions in the study.

The crude prevalence of diagnosed diabetes was 30.3% (95% confidence interval [CI], 25.4%–35.6%) for Indo-Guyanese and 16.1% (95% CI, 12.5%–20.5%) for non-Hispanic whites (*P* < .001) (Table 2). The age-standardized prevalence was 28.7% (95% CI, 23.9%–34.0%) for Indo-Guyanese and 14.5% (95% CI, 10.9%–18.5%) for non-Hispanic whites (*P* < .001), with a prevalence ratio of 2.0. Prevalence increased with age in both groups, and the prevalence ratio between the 2 groups remained relatively constant across age categories. Indo-Guyanese women had significantly higher prevalence than non-Hispanic white women (35.9% vs 14.7%, *P* < .001) and Indo-Guyanese men (35.9% vs 24.2%, *P* = .03). The prevalence of diabetes was nearly 3 times as high among overweight (BMI 25.0–29.9 kg/m^2^) Indo-Guyanese as among overweight non-Hispanic whites (35.9% vs 12.8%, *P* < .001). Family history of diabetes was associated with higher prevalence of diabetes in both Indo-Guyanese and non-Hispanic whites.

**Table 2:** Weighted^a^ Prevalence of Self-Reported Diagnosed Diabetes in Indo-Guyanese and Non-Hispanic White Adults, Schenectady, New York, 2011^b^

Characteristic	Indo-Guyanese		Non-Hispanic White		*P* Value for Prevalence Difference^c^	Prevalence Ratio
	n	% (95% CI)	n	% (95% CI)		
**Crude**	313	30.3 (25.4–35.6)	327	16.1 (12.5–20.5)	<.001	1.9
**Age group, y**						
18–29	72	3.2 (0.5–8.9)	58	1.7 (0.1–8.2)	.99	1.9
30–44	81	12.9 (6.4–20.9)	85	7.7 (3.7–15.6)	.40	1.7
45–59	87	42.9 (32.5–53.1)	98	22.4 (15.0–31.5)	.005	1.9
≥60	73	61.2 (50.1–72.2)	85	28.3 (19.4–38.5)	<.001	2.2
**Sex**						
Male	150	24.2 (17.7–31.3)	159	16.6 (11.2–22.7)	.13	1.5
Female	163	35.9 (29.1–43.8)	168	14.7 (10.6–19.5)	<.001	2.4
**BMI, kg/m^2^**						
<25.0	97	15.5 (9.3–23.7)	115	9.7 (5.1–16.0)	.29	1.6
25.0–29.9	127	35.9 (28.2–44.9)	118	12.8 (7.6–19.7)	<.001	2.8
≥30.0	71	37.5 (27.3–49.7)	84	30.8 (21.8–41.4)	.48	1.2
**Family history of diabetes**						
With family history	178	38.9 (31.8–46.1)	141	24.8 (18.2–32.4)	.01	1.6
No family history	129	18.3 (12.6–26.0)	182	8.1 (4.9–12.9)	.01	2.3
**Age-standardized**	313	28.7 (23.9–34.0)	327	14.5 (10.9–18.5)	<.001	2.0

Abbreviation: CI, confidence interval; BMI, body mass index.

a Data were weighted to reflect the population distributions in the study.

b Numbers may not equal the total because of missing data.

c *z* test for proportions was used to obtain *P* values.

Indo-Guyanese who had diabetes generally had a higher prevalence of diabetes-related complications, comorbidities, and hospitalizations (Table 3). In particular, Indo-Guyanese had a higher prevalence of eye or vision complications (46.4% vs 21.1%, *P* = .004) and were more likely to report their general health status as fair or poor (43.6% vs 15.8%, *P* = .001). Indo-Guyanese who had diabetes had a significantly lower BMI than non-Hispanic whites who had diabetes (28.7 vs 31.2, *P* = .009).

**Table 3:** Weighted^a^ Prevalence of Complications and Health Indicators Among Indo-Guyanese and Non-Hispanic Whites With Diabetes, Schenectady, New York, 2011

Characteristic	Indo-Guyanese		Non-Hispanic White		*P* Value for Prevalence Difference^b^
	n	Value	n	Value	
Age, y, mean (SD)	95	57.6 (12.4)	52	57.9 (14.0)	.89
BMI, kg/m^2^, mean (SD)	87	28.7 (4.3)	52	31.2 (6.9)	.009
Nerve complication, % (95% CI)	94	48.8 (38.9–59.0)	52	36.2 (24.3–50.2)	.20
Eye or vision complication, % (95% CI)	93	46.4 (36.3–56.4)	52	21.1 (11.7–33.8)	.004
Kidney complication, % (95% CI)	90	23.7 (15.5–32.9)	50	15.8 (7.7–28.1)	.38
Diabetes hospitalization, % (95% CI)	93	13.6 (8.0–22.2)	52	9.8 (3.6–20.0)	.69
High blood pressure, % (95% CI)	95	68.1 (58.6–77.2)	53	59.1 (44.9–71.1)	.36
High blood cholesterol, % (95% CI)	95	61.7 (52.1–71.4)	53	67.8 (54.5–79.4)	.57
Heart disease, % (95% CI)	95	23.9 (16.4–33.6)	53	20.1 (11.4–32.2)	.75
Fair or poor general health, % (95% CI)	95	43.6 (33.5–53.3)	53	15.8 (7.3–26.7)	.001

Abbreviation: SD, standard deviation; BMI, body mass index; CI, confidence interval.

a Data were weighted to reflect the population distributions in the study.

b *z* test for proportions was used to obtain *P* values.

## Discussion

Our study is the first community-based study to compare the prevalence of diagnosed diabetes and its complications among Indo-Guyanese adults with the prevalence among non-Hispanic whites. It confirms that diabetes disproportionately affects the Indo-Guyanese community in Schenectady as was suggested in the earlier clinic-based study (2).

This high prevalence of diabetes in people of Indian descent is not unique to Schenectady. It reflects the higher prevalence of diabetes in descendants of Indian diasporas in small islands including Fiji, Trinidad, and Guadeloupe (12–15) as well as Indian immigrants in major cities in the United States (16–18) and Europe (19,20). These consistent observations of higher prevalences of diabetes in generations of Indian immigrants around the world warrant genetic studies. Researchers have reported a constellation of metabolic abnormalities and cardiovascular risks commonly found in populations from the Indian subcontinent, characterized by atherogenic dyslipidemia, glucose intolerance, thrombotic tendency, subclinical inflammation, and endothelial dysfunction (21). One significant finding of our study is that Indo-Guyanese with diabetes have a lower average BMI than non-Hispanic whites with diabetes. In general, Indian populations have higher levels of abdominal visceral fat regardless of their overall adiposity. This “metabolically obese” phenotype (eg, normal weight by conventional BMI standards but increased abdominal adiposity) has been associated with increased risk of insulin resistance and diabetes in Indian populations (17,22). This postulation needs to be studied further in the Indo-Guyanese population.

In addition to genetic predisposition, differences in educational attainment, household income, and insurance status between Indo-Guyanese and non-Hispanic whites suggest differential access to measures that prevent diabetes. Socioeconomic status (SES) is inversely associated with risks of type 2 diabetes, and this association is independent of the income levels of countries (23). Furthermore, rapid transitions in dietary behavior and physical activity status that follow international migration can contribute to the increased diabetes risks in Indo-Guyanese (20,21). Further research is needed to understand the roles of SES (as a confounder or an effect modifier) and lifestyle change in the development of diabetes in this population.

Higher prevalence of eye or vision complications and less favorable ratings of general health suggest poor control of diabetes in Indo-Guyanese. Our preliminary assessment of the Indo-Guyanese community indicated that lack of health insurance coverage, lack of a regular medical care provider, and low health literacy are likely barriers to diabetes control (D.S. Pratt, K.A. Sen, A. Simao Jr, G.S. Hunt, Schenectady County Public Health Services, unpublished data, September 1, 2012). Indo-Guyanese cultural beliefs, attitudes, and collective behavior toward diabetes and its care, as well as social support structure, can also determine diabetes control, but very little research has been conducted to provide sufficient information.

There are limitations in this study. Assessment of diabetes prevalence based on self-reported diagnosis underestimates the true extent of diabetes. The Centers for Disease Control and Prevention reports that approximately 27% of all diabetes cases in the United States are undiagnosed, and 35% of the US population has prediabetes, a major risk factor for type 2 diabetes (24). Undiagnosed diabetes may be more prevalent in Indo-Guyanese than in non-Hispanic whites because Indo-Guyanese have less access to health care. Type of diabetes was not assessed in this study. Almost all cases of diabetes in Indo-Guyanese are believed to be type 2, according to literature and the earlier clinical study (2,20,25). BMI was assessed by self-reported height and weight, which may have resulted in underestimation of BMI. The prevalence of diabetes complications was not adjusted for the duration of diabetes, treatment regimen, or medical care use. We did not collect a measure for abdominal adiposity because of the difficulty of obtaining such information through an interview survey.

There are also intrinsic limitations of convenience sampling, including inability to generalize its findings. It is likely that people affected by diabetes were more willing to participate in the survey, resulting in volunteer bias. High socioeconomic status of the non-Hispanic white sample could be an indication of selection bias as well. We do not know how age, sex, and socioeconomic characteristics differ between Indo-Guyanese and non-Hispanic white populations because of the lack of relevant census information. Given that random-digit–dialing telephone methods also have intrinsic limitations for reaching low SES immigrant minority groups like Indo-Guyanese (26), we believe our sampling strategy was appropriate for our study objective.

This community-based study found nearly twice the overall prevalence of diagnosed diabetes in Indo-Guyanese adults than in non-Hispanic white adults. Indo-Guyanese also had signs of poor diabetes control. The disproportionate burden of diabetes in the Indo-Guyanese population could be larger if undiagnosed cases and prediabetes are taken into account. Although the Indo-Guyanese population is most likely genetically predisposed to develop diabetes, low SES, immigration experience, and cultural factors are also likely to be associated with the higher burden of diabetes. Further research to understand the complex mechanism of diabetes disparities is strongly needed. Medical professionals should be aware that Indo-Guyanese adults can develop type 2 diabetes without becoming obese. Diabetes screening criteria specific to South Asian populations may be more appropriate for assessing diabetes risks in the Indo-Guyanese population (27).
